# Sleep Quality and Eating Disorder-Related Psychopathologies in Patients with Night Eating Syndrome and Binge Eating Disorders

**DOI:** 10.3390/jcm10194613

**Published:** 2021-10-08

**Authors:** Orna Tzischinsky, Itay Tokatly Latzer, Sigal Alon, Yael Latzer

**Affiliations:** 1 Behavioral Science and the Center for Psychobiological Research, Emek-Yezreel Academic College, Emek Yezreel 19300, Israel; orna3007@gmail.com; 2 Pediatric Neurology Institute, Dana-Dwek Children’s Hospital, Tel Aviv Sourasky Medical Center, Tel Aviv 6423906, Israel; itaylatzer@gmail.com; 3 Sackler Faculty of Medicine, Tel Aviv University, Tel Aviv 6997801, Israel; 4 Eating Disorders Institution, Psychiatric Division, Rambam Health Care Campus, Haifa 31096, Israel; s_alon@rambam.health.gov.il; 5 Faculty of Social Welfare and Health Sciences, University of Haifa, Haifa 3498838, Israel

**Keywords:** night eating syndrome, bulimia nervosa, binge eating disorders, sleep quality, actigraph, psychopathology

## Abstract

Night eating syndrome (NES) is an eating disorder (ED) characterized by nocturnal ingestion (NI), evening hyperphagia, morning anorexia, as well as mood and sleep disturbances. This study compared subjective and objective sleep quality and ED-related psychopathologies in patients seeking treatment for ED. Method: The sample was composed of 170 women, aged 18–68, who were referred for an ED assessment from 2011 to 2020. The participants were divided into three subgroups: NES-NI only (*n* = 30), NES+ binge eating (BE) (including binge eating disorders or bulimia nervosa (*n* = 52), and BE-only (*n* = 88). The measures consisted of a psychiatric evaluation, objective sleep monitoring measured by an actigraph for 1 week, a subjective sleep self-report, and ED-related psychopathology questionnaires. Results: Objective sleep monitoring revealed significant group differences, with higher sleep efficiency in participants with BE-only and longer sleep durations for the NES-NI only group. Subjectively, the BE-only group described a significantly lower sleep quality than either the NES-NI only or the NES+BE groups. ED-related psychopathology was lower in the NES-NI-only group. A stepwise linear regression revealed that general psychopathology (the brief symptom inventory total score) was a significant predictor of subjective sleep quality. **Conclusion:** NES-NI-only was correlated with less psychopathology, but with more subjective and objective sleep disturbances. These results lend weight to the supposition that NES lies on a continuum of ED psychopathologies, and that NES-NI-only appears to be a separate entity from NES+BE and BE-only in terms of its psychopathology.

## 1. Introduction

Night eating syndrome (NES) is a clinical entity characterized by both eating and sleeping disorders. NES was initially described by Stunkard in 1955 and is characterized by night ingestion (NI), evening hyperphagia (EH), morning anorexia, insomnia, declining mood that worsens in the evening, and emotional distress [1].

An international interdisciplinary group composed of sleeping and eating disorder experts published diagnostic criteria for NES in 2010 [2]. These diagnostic criteria were then generally included in the DSM-5 [3] under “Other specified feeding and eating disorders.”

Studies have shown that NES is more common in individuals with overweight or obesity. Findings indicate that the prevalence of NES in an adult community sample was approximately 1.5% [4,5,6], 8.9% in individuals seeking weight-loss treatment, [4,5], and 9% and 16% in patients seeking treatment for ED such as bulimia nervosa and binge eating disorder, respectively [7]. In patients with a psychiatric diagnosis excluding psychosis, bipolar, substance use or eating disorders, the prevalence rates of NES was reported to range from 12.3% to 22.4% [8,9].

In a recent study using the DSM-5 diagnostic criteria for NES [3] the prevalence rates were 51.7% for binge eating disorders and 34.9% for bulimia nervosa [10]. Only a few studies have examined NES in patients diagnosed with EDs. Studies that have examined NES in patients with ED [7,11,12,13] have mostly focused on individuals with BED [14,15,16] or bulimia nervosa (mainly case studies) [10,15,17,18,19,20,21,22,23]. The prevalence rates tend to vary considerably across studies since the diagnostic criteria for NES were frequently modified until the new criteria were defined in the DSM-V [3].

Binge eating (BE) behavior is a clinical presentation covering binge eating disorders, bulimia nervosa, and NES, which may be accompanied by binge episodes and a feeling of lack of control [3]. They differ, however, in two major ways. Individuals with BN exhibit compensatory behaviors after binge episodes, unlike individuals with BED or NES. Individuals with BED or BN commonly have binge episodes throughout the day, whereas individuals with NES typically engage in binge eating during the evening or after sleep onset [2,3]. The prevalence of psychiatric comorbidity was found to be high in patients with BN [24], BED [25], and NES [14,26].

The sleep-related eating disorder (SRED) is considered to relate to sleep disorders rather than to eating disorders. SRED and NES both include NI, and although the differences between SRED and NES are somewhat controversial, it is useful to differentiate between them [27]. SRED is considered to be a sleep disorder (parasomnia) whereas NES is considered to be an eating disorder. The level of consciousness during NI episodes is the main differentiating factor between them: SRED is characterized by a lack of awareness during the night eating episodes whereas NES takes place during full consciousness [28]. For this reason, SRED was excluded from the new NES diagnostic criteria in the DSM-5, as it was considered a unique diagnosis representing a sleep disorder and not an ED [2,14]. Despite this classification, the relationships between NES, ED, and SRED still elicit debate in the literature [11,14,27,29,30].

Sleep disturbances in individuals with EDs are well-documented. Studies suggest that ED pathologies tend to be associated with psychiatric comorbidity and insomnia, with stronger associations as the severity of the ED symptoms increases. One report indicated that about 5% of the healthy participants experienced symptoms of insomnia, whereas insomnia was found for 14% of the participants with a high risk of developing ED, and 25% to 30% of those with a diagnosed ED. [31].

Low sleep quality in individuals with BED has been linked to a high risk of binge eating, even after controlling for obesity and depression [32,33]. In individuals with BN, poor sleep was accompanied by late sleep onset [34]. Individuals with bulimia nervosa symptoms and social pressures related to eating were found to have more sleep disturbances [35].

No differences in patients with NES were found for sleepiness in overweight night eaters (body mass index [BMI] < 25.0 kg/m^2^) and controls, suggesting that patients with NES do not experience excessive daytime sleepiness. However, patients with NES reported a significantly higher rate of sleep disturbances, took more sleeping pills, and had less satisfactory daytime functioning, according to the Pittsburgh Sleep Quality Index (PSQI; [36,37,38]). In addition, severe obesity (BMI ≥ 35 kg/m^2^) with NES was associated with more sleep disturbances, and feelings of insomnia [39]. Studies implementing actigraphy and self-report sleep diaries while comparing sleep-wake cycles between females with and without NES have shown that patients with NES woke up early during the night, had more awakenings and woke up later in the morning [38,40]. Polysomnographic recordings indicated that the number of awakenings to eat varied among individuals but usually occurred during non-rapid eye movement (non-REM) sleep, whereas only a few awakenings took place during rapid eye movement (REM) sleep [14]. The time spent awake to eat was typically brief, and patients fell asleep quickly after eating [37,41]. However, only a few studies have examined subjective and objective sleep disturbances in terms of the level of psychopathology in patients with NES and ED who seek treatment for ED [7,10,38].

To respond to this need, the current study had four objectives: 1. To compare subjective self-reported and objective (as measured by actigraphy) sleep quality in three groups of patients: NES-NI-only, NES+BE (NES+BED or BN), and BE-only (including BED or BN); 2. To compare the ED-related psychopathology in these three groups; 3. To examine the relationship between the objective and subjective quality of sleep and ED-related psychopathology in the three groups; 4. To predict the objective and subjective sleep quality based on ED-related psychopathology, with the groups serving as potential moderators.

## 2. Method

### 2.1. Participants

The sample was composed of 170 women aged 18–68 years [mean ± standard deviation (SD) 36.5 ± 13.3] who were referred to an ED institution in Israel for an ED assessment between 2011 and 2020.

The exclusion criteria were individuals with SRED (based on a diagnosis of a sleeping disorder) [42], AN (low prevalence) [7] or other severe psychiatric illnesses. Twenty-seven patients (14%) were either excluded or dropped out of the study (incomplete questionnaires or withdrew).

The participants were recruited at admission prior to treatment and were divided into the three subgroups, NES+-BE, NES-NI- only, and BE-only, as follows:

(a) NES+BE (*n* = 52) included individuals diagnosed with NES (NI, EH, or both), [2,43] who also met the full diagnostic criteria for either binge eating disorder (*n* = 34) or bulimia nervosa (*n* = 18) according to the DSM-IV (since the study started before the DSM-5) [2,43];

(b) NES-NI only (*n* = 30) included individuals diagnosed with NES-NI-only based on the DSM-5 and [2,3], without binge eating disorders or bulimia nervosa (see Table 1);

(c) BE-only (*n* = 88) included individuals with binge eating disorders (*n* = 49) or BN (*n* = 39), according to the DSM-IV [43].

Comparisons were conducted between patients with bulimia nervosa and patients with binge eating disorders on the objective and subjective quality of sleep and ED-related psychopathology. *T*-tests indicated no significant differences between bulimia nervosa and binge eating disorders for most variables (and on a Mann–Whitney test for sleep efficiency) except, as expected, for age and BMI (weight [kg] divided by height squared [m^2^]) and wake-up time (after covariate for age; [44]. Therefore, bulimia nervosa and binge eating disorders were merged into one subgroup for further analyses, labeled BE-only (see Table 1).

### 2.2. Procedure

This study was approved by the s Ethics Committee of a medical center in the northern part of Israel (40-09RAM). The participants provided signed informed consent.

Data were gathered during evaluation meetings. Patients underwent a full clinical psychiatric assessment using the mini international neuropsychiatric interview (MINI-SCID; [45] for bulimia nervosa and binge eating disorders (DSM-IV; [43], and for NES, according to the recommended diagnostic criteria for NES [2,3].

The diagnostic criteria recommended for NES were as follows: significantly increased (at least 25% of food intake) eating in the evening after dinner (EH) or waking up to eat during the night (NI), at least twice a week, and awareness of eating. In addition, at least three of the following characteristics were mandatory: morning anorexia, a desire to eat after dinner and after sleep onset, or in the middle of the night, insomnia, a belief that one should eat in order to fall asleep, and a depressed mood mainly during the evening. For inclusion, NES needed to have been linked to substantial emotional distress, for at least three consecutive months, and not be due to medical or other psychiatric disorders. After completing the psychiatric assessment, age, sex, weight, and height information was retrieved.

Patients who agreed to participate in the study filled out self-report questionnaires and were given an actigraph to wear on their non-dominant wrist to monitor sleep for at least 5 days and nights. After returning the actigraph and the self-reported sleep questionnaire, the principal investigator analyzed sleep quality using the AW2 program [46]. Thereafter, each participant was sent an individual report with specific recommendations for further sleep evaluation or treatment as needed.

### 2.3. Measures

#### 2.3.1. Demographic and Clinical Data

Participants provided age, height, and weight data.

#### 2.3.2. ED Diagnosis

The MINI-SCID is a structured diagnostic interview used to investigate 17 disorders corresponding to the DSM-IV. Comparison of the MINI-SCID with clinical psychiatric assessment according to DSM-IV for eating disorder diagnosis showed good sensitivity (0.92), and specificity (0.99) [45].

#### 2.3.3. Subjective Sleep Quality

The Pittsburgh Sleep Quality Index (PSQI) [47,48] is a self-report questionnaire assessing sleep during the previous month prior to participation. It is intended to assess sleep quality in individuals from clinical or communal settings. The PSQI is composed of 19 items: 15 are ranked from 0 to 3, whereas 4 are open-ended questions that are used for a quality evaluation of sleep quality as perceived by the participant. These 19 items are used to generate 7 subscales and a total score. A total score over 5 suggests insufficient sleep quality. The reliability of this measure for this study was acceptable (Cronbach’s alpha= 0.79).

#### 2.3.4. Objective Sleep Quality

Objective sleep quality was assessed by actigraph (Mini-Act, AMA-32, AMI), a small movement sensor that continuously records body motility data for long periods of time. The actigraph accumulates movements in bins of 1 min which cause the sensor signal to cross a fixed reference signal. Sleep/wake measures were estimated from actigraphy data using a validated algorithm. [49]. The actigraph was worn on the wrist of the non-dominant hand for a 5-night period, based on a previous study that indicated that 5 nights were sufficient for sleep quality measures [49]. Use of the actigraph for both research and clinical purposes under natural conditions has been reported in previous studies to have adequate reliability and validity.

Overall agreement rates with polysomnographic scoring range from 91% to 93% for the calibration and validation samples. Previous studies indicate that the actigraphs vary considerably in their sensitivity. Nevertheless, the validation results indicated that for normal subjects the agreement rates between the actigraph-based and polysomnographic recording, based minute-by-minute sleep wake scoring were above 90% [50]. The actigraph measures sleep onset time, wakeup time, sleep efficiency, and sleep duration [51].

#### 2.3.5. ED-Related Psychopathology (Self-Report)

**The Beck Depression Inventory** (BDI) [52] is a 21-item inventory that addresses symptoms of depression during the previous week. It specifically assesses physical, emotional, and cognitive aspects of depression. Each item is ranked from 0 to 3. The total score provides an efficient summary of the participant’s depression level, with scores > 10 indicating mild depression, >20 indicating moderate depression, and >30 indicating severe depression. The Hebrew version was assessed and validated in ED populations [53,54]. The Cronbach’s alpha for the Hebrew version was 0.87.

**The Eating Disorder Inventory** (EDI-2) [55] is a self-report questionnaire addressing ED related to psychopathology. EDI-2 assesses a profile of symptoms frequently found in patients with ED but is not designed to be used as a diagnostic tool. The EDI-2 is composed of 91 items, scored from 0 to 3. The EDI-2 contains 11 subscales with total scores ranging from 0 to 273, with higher scores implying more ED-symptoms related to psychopathology. The EDI-2 is a valid and reliable tool in many settings and languages, including Hebrew [56]. In the current study, we used the EDI total score. The Cronbach’s alpha for the Hebrew version was 0.87.

**The Brief Symptom Inventory** (BSI) [57] consists of 53 items and is a self-report questionnaire assessing the psychological state of patients seeking psychiatric and other treatments. The BSI is rated from 0 to 4 with a high score indicating a high frequency. In the current study, only the total score was used, with a higher score indicating more psychiatric symptoms. The BSI is a valid and reliable tool in many settings and languages, including Hebrew [58]; the Cronbach’s alpha for the Hebrew version was 0.96.

**The Spielberg State-Trait Anxiety Inventory** (STAI) [59] is a self-report questionnaire that evaluates anxiety level. This questionnaire is composed of 40 items; 20 items evaluate the state of anxiety (the level of anxiety while testing-STAI-S), and the other 20 items evaluate anxiety as a trait (the general tendency to develop anxiety STAI-T). Participants report state and trait anxiety according to their feelings, on a scale of 1 to 4. Low scores indicate calmness and confidence, whereas high scores reflect high anxiety, stress, and worry. Several items are reverse-scored. The general score range is 20 to 80. The questionnaire was translated and validated in Hebrew for general populations [60] and also for ED populations [54]. The reliability of the STAI-State and STAI-Trait scales in this study was excellent (Cronbach’s alpha 0.92 and 0.93, respectively).

**The Body Shape Questionnaire** (BSQ) [61] is a 34-item self-report questionnaire assessing body shape concerns, self-depreciation related to physical appearance, and the cognitive experience of feeling fat. In the current study we used the total score (ranging from 34 to 208). Higher scores (above 98) represent a lower body image. The reliability in this study was excellent (Cronbach’s alpha = 0.97).

**The Social Phobia Inventory** (PHI) [62] is a 17-item self-report questionnaire, assessing the severity of social anxiety disorder and social phobia. In the current study we used the total scores (ranging from 17 to 85). Higher score indicate greater social phobia. The reliability in this study was excellent (Cronbach’s alpha = 0.90).

### 2.4. Data Analysis

Data analyses were performed using SPSS-25 (IBM SPSS Statistics, 2017, IBM Corp, Armonk, NY, USA). Normality testing and descriptive statistics were analyzed for the entire sample. The variables for age, BMI, BDI, STAI-T, STAI-S, body image, and BSI presented a normal distribution (using skewness and kurtosis to be ± 1.5 (we had ± 1.1)). Sleep onset time and sleep efficiency were not normally distributed; however, when two outliers were removed (more than 3 SD from the mean; the patients fell asleep after 4 a.m.), sleep onset hour was normally distributed. Thus, only sleep efficiency needed to be analyzed with nonparametric statistics.

Differences in objective and subjective sleep quality and ED-related psychopathology in the groups were tested using an analysis of variance for normally distributed data and the Kruskal Wallis test for non-normally distributed data to compare the three groups (BE-only, NES+BE, and NES-NI-only). Pearson or Spearman correlations, where appropriate, were performed to assess the relationships between ED-related psychopathology and subjective and objective sleep patterns. In order to adjust for multiple comparisons, the false discovery rate was computed according to Benjamini and Hochberg (1995) [63]. To predict sleep quality (subjective and objective) based on ED-related psychopathology, a linear regression analysis was conducted with group serving as a potential moderator. Group was considered a significant moderator if the interaction between group and the independent variable was significant.

Cohen’s d was computed as a measure of effect size for the comparison between BED only and BN only where 0.2 was a small effect size, 0.5 a medium effect size and 0.8 and above a large effect size. Partial eta squared was computed as a measure of effect size for the three-group comparison. Here, 0.01 was considered to be a small effect size, 0.06 a medium effect size, and 0.014 or higher a large effect size.

## 3. Results

The results reflect comparisons among the three groups: NES-BE-only, BE-only, and NES-NI-only for objective and subjective sleep quality and ED-related psychopathology.

A univariate analysis showed significant differences in age between the BE-only and the other groups, where individuals in the BE-only group were significantly younger than those in the NES-NI-only group (mean difference = −11.7 years, SE = 2.6, *p* < 0.001) and NES + BE group (mean difference= −7.8 years, SE = 2.2, *p* < 0.001). Statistically significant differences were found for the total BSI score, social phobia, and body image; Post hoc analyses revealed that the NES-NI-only group had a significantly lower total BSI score than the NES-BE group (mean difference = −0.53, SE = 0.17, *p* < 0.006). In addition, the NES-NI-only group had significantly lower social phobia and body image scores than the NES+BE group (mean difference = −16.00, SE = 5.28, mean difference = −39.42, SE = 13.51, *p* < 0.009, *p* < 0.01, respectively) and BE-only group (mean difference = −16.67, SE = 4.94, *p* < 0.003; mean difference = −38.28, SE = 12.70, *p* < 0.01, respectively) groups.

A univariate analysis of the subjective and objective sleep variables revealed a statistically significant difference in PSQI-total score, where the BE-only group had significantly lower scores than the NES+BE group (mean difference = −2.05, SE = 0.58, *p* < 0.002, mean difference −7.56, SE = 1.75, *p* < 0.001, respectively). Sleep onset time was about 52 min later in the BE-only group compared to the NES-NI-only group (mean difference = 0.87, SE = 0.28, *p* < 0.007). This remained true when the two outliers were removed (mean difference = 0.82, SE = 0.26, *p* < 0.006). Sleep duration was 36 min longer in the NES-NI-only group compared to the NES+BE group (mean difference = 36.4, SE = 15.1, *p* < 0.05). The BE-only group had a statistically significantly higher median sleep efficiency than the NES-NI-only (*p* < 0.003) and NES+BE (*p* < 0.003) groups. After adjusting for age, group differences persisted. However, there was no longer a statistically significant difference in sleep onset time between the 3 groups (see Table 2).

Table 3 shows significant correlations between PSQI-total and BDI (0.259, *p* < 0.001), and BSI-total (0.398, *p* < 0.001), whereas poorer sleep (PSQI > 5) was correlated with higher depression (BDI), and general psychopathology (BSI). However, no significant correlation was found between objective sleep quality (as measured by the actigraph) and any of the psychopathology measures (see Table 3).

In the NES+BE group, BDI and BSI-total were positively correlated with PSQI-total (0.528, *p* < 0.001; 0.633, *p* < 0.001, respectively). In the NES-NI-only group, BSI-total was positively correlated with PSQI-total (0.534, *p* < 0.01). In addition, age was negatively correlated with wakeup time (−0.428, *p* < 0.001).

After adjusting for age, BSI-total (F (1, 132) =26.60, *p* < 0.01, partial eta = 0.17) and ED groups (F(2, 132) = 4.08, *p* < 0.02, partial eta = 0.06) were significant predictors of PSQI-total. Bonferroni post- hoc testing revealed that the BE group tended to have lower PSQI-total scores than the NES-NI-only (mean difference = −1.68, SE = 0.71, *p* < 0.06) and NES+BE (mean difference = −1.34, SE = 0.58, *p* < 0.065) groups. For every 1-point increase in BSI, PSQI increased by 1.7 points (*b* = 1.72, SE = 0.33, 95% CI = 1.06, 2.38). Group was not a significant moderator of BSI-total (F(2, 130) = 2.95, *p* > 0.06). ED- related psychopathology (BDI, BSI-total, social phobia, and body image) was not a statistically significant predictor of objective sleep efficiency.

## 4. Discussion

Night eating syndrome is a proposed ED diagnosis that characterized mainly by NI, EH, morning anorexia, mood, and sleep disturbances, as noted in the DSM-5 [3] and Alison et al. 2010 [2]. ED psychopathology is related to psychiatric comorbidity and sleep disturbances, particularly among patients with EDs [31] and NES [38]. To the best of our knowledge, only a few studies have examined objective and subjective sleep efficiency in conjunction with psychopathology in patients with ED and NES. This study compared objective and subjective sleep efficiency and ED as related to psychopathology in individuals treated for ED.

Subjective sleep quality (based on PSQI) indicated that all the ED groups reported poor sleep quality (PSQI > 5); nevertheless, both the NES+BE and NES-only groups had significantly poorer subjective sleep quality than the BE-only group. This finding may be related to the fact that NES-NI patients wake up a few times after sleep onset in order to eat. These awakenings may affect sleep quality that can lead to a subjective feeling of poorer sleep when waking up in the morning. These results are similar to previous findings on smaller sample sizes, which reported low sleep quality among patients with NES [7,14], and works that have found low subjective sleep quality among patients with BN-only, BN and NES, and NES-NI-only [10]. However, none of these studies assessed patients with BED or used objective sleep assessments.

Objective sleep quality, as measured by the actigraph, revealed significant differences among the ED groups, where the BE-only group had higher sleep efficiency than either the NES+BE and NES-only groups. In addition, the NES-only group had a significantly earlier sleep onset (about 30 min) and about a 30 min longer sleep duration than the BE-only and NES-BE groups. One explanation for the longer sleep duration in the NES-only group may be related to the several awakenings during the night of individuals with NI. They may sleep longer in order to compensate for their sense of sleep deprivation, as suggested elsewhere [7,38]. A recent study using polysomnography found that individuals with NES-NI showed objective sleep disturbances, such as a decrease in sleep efficiency and duration, that may be a result of sleep disturbances [14].

The significant group differences in sleep onset time, where the NES-only group fell asleep about 30 min earlier than either the BE-only or the NES+BE groups, may be related to the greater number of binge eating episodes occurring mainly after dinner throughout the evening in patients with binge eating symptoms (BN or BED) and with NES-EH [33,34,64].

There was a high level of psychopathology in all three ED groups. The NES-only group had the lowest psychopathology, as reflected in their statistically significantly different BSI, body image (BSQ), and social phobia (PHI) scores and marginally significant differences in disordered eating psychopathology (EDI-2) and depression (BDI). There were no significant differences between the NES+BE and BE-only groups in terms of psychopathology. These results are consistent with previous findings indicating higher levels of psychopathology among patients with BED or BN [65,66] and patients with NES+BE [10,12,24,26,36,67]. Thus, further research is needed to differentiate between BED, BN with and without NES, and between BED, BN, and NES-NI and NES-EH.

The analyses also revealed significant correlations between subjective sleep disturbances (PSQI), depression (BDI), and general psychopathology (BSI), in that subjectively poor sleep quality was associated with higher rates of depression and general psychopathology. However, no significant correlation was found between objective sleep quality and any of the psychopathology measures. These results are in line with studies indicating discrepancies between objective and subjective sleep disturbances in patients with AN [68], BN [34], BED [33], and NES [7,14,38], and may relate to the level of psychopathology rather than to objective sleep disturbances. These results are also supported by recent studies indicating that patients with BED report significantly poorer subjective sleep than healthy controls with no ED [69] and a report indicating that ED in young adults predicts subjective sleep disorders at a 7-year follow-up [70]. However, these studies did not measure objective sleep quality.

A stepwise linear regression revealed that general psychopathology (BSI) was a significant predictor of subjective sleep disturbances (PSQI) and explained 15.2% of the variance. This strengthens the assumption that subjective sleep quality is associated with the level of psychopathology rather than objective sleep quality, and is in line with previous results on patients with ED [33,34,38,68]. The discrepancies between subjective complaints of sleep disorders and electroencephalogram (EEG) findings are commonly seen in studies on patients with post-traumatic stress disorder (PTSD) [71,72]. These may be related to the traumatic life events associated with ED in general [13] and ED with NES in particular [12].

### Limitations and Future Directions

A number of limitations should be taken into consideration. A larger sample size would have better differentiated between NES-NI and NES-EH, and the identification of possible differences in psychopathology and sleep patterns between groups. It is important to note that BED and NES-EH with binge episodes during the evening may meet the dual diagnostic criteria of both BED and NES and may confuse clinicians. Therefore, further research is needed to justify classification of NES-EH and NES-NI, given that NES currently includes both, since NES-EH is similar to BED and may be part of this diagnosis, whereas NES-NI could be a separate diagnosis.

Secondly, an actigraph was used to evaluate objective sleep patterns. This procedure has been widely used in the last 30 years in studies estimating objective sleep patterns in populations with ED. However, it cannot describe all objective sleep disturbances; hence, further research with more detailed measurements such as polysomnographic recordings of sleep disturbances and sleep stages is suggested to further identify similarities and differences in sleep–wake cycles among ED groups.

Finally, the current sample consisted of patients who were seeking treatment for ED, and who may tend to exhibit higher levels of psychopathology than those seeking treatment in non-clinical centers, usually for weight loss. Further research is needed on individuals with NES in a nonclinical sample that may help account for the differences between patients with ED and NES. It may also better situate NES on the ED psychopathology spectrum.

## 5. Conclusions

The current study assessed objective and subjective sleep quality and level of ED-related psychopathology in patients seeking treatment for ED (BN or BED with and without NES and NES-NI only) using the newly suggested diagnostic criteria for NES [2,3]. It also included new self-report measures of NES-NI.

The NES groups (NES+BE and NES-NI) showed significantly poorer sleep quality and had significantly worse sleep efficiency than the BE-only group. In terms of psychopathology, body image, and social phobia were significantly lower in the NES-NI-only group. Subjective sleep quality was predicted by the level of psychopathology (BSI) and the ED group, in that subjective (vs. objective) sleep quality was associated with an increased level of psychopathology, mainly in the NES+BE and NES-NI groups.

Overall, these results highlight the importance of a clearer clinical grasp and early evaluation of NES, subjective sleep disturbances, and levels of psychopathology in individuals with ED.

## Figures and Tables

**Table 1:** Comparison of BN and BED in terms of objective and subjective quality of sleep and ED-related psychopathologies (mean ± sd; range).

	BED Only (*n* = 49)	BN Only (*n* = 39)	t(df); *p*	Effect Size
Age	34.69 ± 11.73 20–68	26.74 ± 7.72 18–53	3.685 (81); <0.001	0.801
BMI	32.79 ± 6.62 20.4–46.7	23.78 ± 4.70 18.2–36.5	7.081 (78); <0.001	1.569
***ED psychopathology***				
BDI	15.79 ± 10.89 0–36	21.00 ± 13.19 0–52	−1.846 (71); n.s.	0.423
EDI total	91.42 ± 31.17 34–164	96.03 ± 41.59 32–198	−0.535 (70); n.s.	0.150
BSI total	1.44 ± 0.67 0.02–2.90	1.55 ± 0.65 0.5–3.2	−0.706 (69); n.s.	0.167
SPIL-S	53.90 ± 11.26 23–73	53.76 ± 12.39 35–77	0.041 (68); n.s.	0.032
SPLI-T	54.90 ± 12.20 26–75	56.24 ± 12.37 33–79	−0.383 (68); n.s.	0.144
Social phobia	43.90 ± 12.13 0–73	40.33 ± 12.94 25–69	0.746 (68); n.s.	0.264
Body image	132.19 ± 41.61 0–199	130.81 ± 34.94 63–184	0.125 (50); n.s.	0.039
***Subjective Sleep***				
PSQI total	8.56 ± 3.23 3–16	8.67 ± 2.55 5–14	−0.148 (70); n.s.	0.118
***Objective Sleep***				
Sleep onset time	24.10 ± 0.88 23.0–26.6	24.64 ± 1.46 21.8–28.7	−1.887 (69); n.s.	0.433
Wake-up time	7.32 ± 1.07 4.77–10.25	8.17 ± 1.26 5.9–10.9	−3.084 (70); <0.05	0.768
Sleep duration	438.79 ± 51.42 310–538	448.65 ± 70.6 309–597	−0.670 (70); n.s.	0.160
Sleep efficiency	93.14 ± 4.33 80.1–99.0	92.22 ± 9.24 58–99	0.34 (70); n.s.	0.128

**Table 2:** Comparison of ED subgroups in ED-related psychopathology and objective and subjective sleep patterns.

	BE-Only	NES-Only	NES+BE				
	(*n* = 88)	(*n* = 30)	(*n* = 52)				
	*M* ± *SD*	*M* ± *SD*	*M* ± *SD*	F (df1, df2)	*p*	Effect Size	Adjusted *p*
Age	32.0 ± 11.6	43.7 ± 12.8	39.8 ± 13.5	12.64 (2, 169)	<0.001	0.130	--
BMI	29.01 ± 7.38	30.14 ± 6.69	31.88 ± 7.16	2.52 (2, 161)	0.08	0.030	--
***ED psychopathology***							
BDI	18.14 ± 12.15	15.43 ± 11.37	22.24 ± 11.59	2.92 (2, 144)	0.06	0.040	0.01
EDI-2	92.96 ± 36.45	77.09 ± 40.69	99.93 ± 33.11	2.96 (2, 140)	0.06	0.041	0.06
BSI-total	1.50 ± 0.66	1.20 ± 0.80 *	1.73 ± 0.76	4.97 (2,158)	0.008	0.059	0.009
SPIL-S	53.53 ± 11.86	49.38 ± 5.42	53.59 ± 9.57	0.88 (2,97)	0.42	0.018	0.64
SPLI-T	55.13 ± 12.38	48.38 ± 8.18	56.62 ± 9.63	2.62 (2, 97)	0.08	0.051	0.12
Social phobia	42.98 ± 16.77	26.31 ± 17.07 *	42.31 ± 14.27	5.95 (2, 98)	0.004	0.108	0.02
Body image	129.89 ± 37.69	91.62 ± 51.27 *	131.03 ± 42.65	5.00 (2, 99)	0.009	0.092	0.03
***Subjective sleep***							
PSQI-total	8.57 ± 2.91 *	10.09 ± 2.93	10.62 ± 3.07	7.05 (2, 136)	<0.001	0.094	0.003
***Objective sleep***							
Sleep onset time	24.36 ± 1.19	23.48 ± 1.31 *	23.98 ± 1.22	5.09 (2, 144)	0.007	0.066	0.08
Wakeup time	7.68 ± 1.21	7.32 ± 1.22	7.19 ± 1.04	2.78 (2, 145)	0.07	0.037	0.46
Sleep duration	441.02 ± 61.27	470.20 ± 65.39 *	433.76 ± 58.59	3.04 (2, 145)	0.05	0.040	0.02
Sleep efficiency (median ± IQR)	95.0 ± 6.0 *	90.6 ± 8.8	89.7 ± 8.0	χ^2^ (2) =13.24	<0.001	0.078	0.02

Note. IQR = interquartile range. Effect size is partial eta squared. * significant after post hoc test (*p* < 0.05).

**Table 3:** Pearson correlation coefficients between ED-related psychopathology and objective and subjective sleep quality (Spearman correlations with efficiency).

	PSQI-Total	Onset	Wakeup	Duration	Efficiency
BDI	0.259 *	−0.018	0.011	0.016	0.116
EDI-2	0.212 *	−0.070	0.040	0.128	0.161
BSI-total	0.398 **	−0.003	−0.003	−0.008	0.163
SPIL-S	0.144	0.064	0.139	0.066	0.107
SPLI-T	0.182	0.080	0.246 *	0.158	0.117
Social phobia	0.077	0.060	0.028	−0.021	0.148
Body image	−0.010	−0.099	0.031	0.140	0.158
Age	0.110	−0.275 **	−0.389 **	−0.117	−0.250 *
BMI	0.048	−0.079	−0.237 *	−0.167 *	−0.161

* *p* < 0.01. ** *p* < 0.001.
